# Design of an improved universal signal peptide based on the α-factor mating secretion signal for enzyme production in yeast

**DOI:** 10.1007/s00018-021-03793-y

**Published:** 2021-03-09

**Authors:** Pablo Aza, Gonzalo Molpeceres, Felipe de Salas, Susana Camarero

**Affiliations:** Centro de Investigaciones Biológicas Margarita Salas, CSIC, Ramiro de Maeztu 9, 28040 Madrid, Spain

**Keywords:** Signal peptide, Synthetic design, Enzyme heterologous expression, Α-factor preproleader, *Saccharomyces cerevisiae*, Directed evolution

## Abstract

*Saccharomyces cerevisiae* plays an important role in the heterologous expression of an array of proteins due to its easy manipulation, low requirements and ability for protein post-translational modifications. The implementation of the preproleader secretion signal of the α-factor mating pheromone from this yeast contributes to increase the production yields by targeting the foreign protein to the extracellular environment. The use of this signal peptide combined with enzyme-directed evolution allowed us to achieve the otherwise difficult functional expression of fungal laccases in *S. cerevisiae*, obtaining different evolved α-factor preproleader sequences that enhance laccase secretion. However, the design of a universal signal peptide to enhance the production of heterologous proteins in *S. cerevisiae* is a pending challenge. We describe here the optimisation of the α-factor preproleader to improve recombinant enzyme production in *S. cerevisiae* through two parallel engineering strategies: a bottom-up design over the native α-factor preproleader (α_nat_) and a top-down design over the fittest evolved signal peptide obtained in our lab (α_9H2_ leader). The goal was to analyse the effect of mutations accumulated in the signal sequence throughout iterations of directed evolution, or of other reported mutations, and their possible epistatic interactions. Both approaches agreed in the positive synergism of four mutations (Aα9D, Aα20T, Lα42S, Dα83E) contained in the final optimised leader (α_OPT_), which notably enhanced the secretion of several fungal oxidoreductases and hydrolases. Additionally, we suggest a guideline to further drive the heterologous production of a particular enzyme based on combinatorial saturation mutagenesis of positions 86th and 87th of the α_OPT_ leader fused to the target protein.

**Supplementary Information:**

The online version contains supplementary material available at 10.1007/s00018-021-03793-y.

## Introduction

Enzyme heterologous expression has been a target of interest in protein research for the last decades. Historically, the yeast *Saccharomyces cerevisiae* has served as an expression system for eukaryotic proteins since it meets the essential protein post-translational demands, such as formation of disulfide bonds or glycosylation [1, 2] and, besides, its simple growth requirements, low average production time and well-known genome facilitate its manipulation [3, 4]. From and industrial point of view, *S. cerevisiae* is still extensively used for obtaining therapeutic proteins [5] and pharmaceuticals products approved for human use by the Food and Drug Administration (FDA) [6]. However, very often, the protein yields provided by this yeast is barely sufficient for research and certainly not suitable for commercialization [7, 8]. The use of yeast signal peptides has been one of the most successful strategies utilized so far to enhance secretion of recombinant enzymes, since signal peptides determine the secretion pathway of the proteins and traffic them to their final site of action [9, 10]. Among them, the leader sequence of the α-factor mating pheromone of *S. cerevisiae* (MFα1) has played an important role in the production of recombinant proteins from different sources in yeast [11–14].

**Fig. 1:**
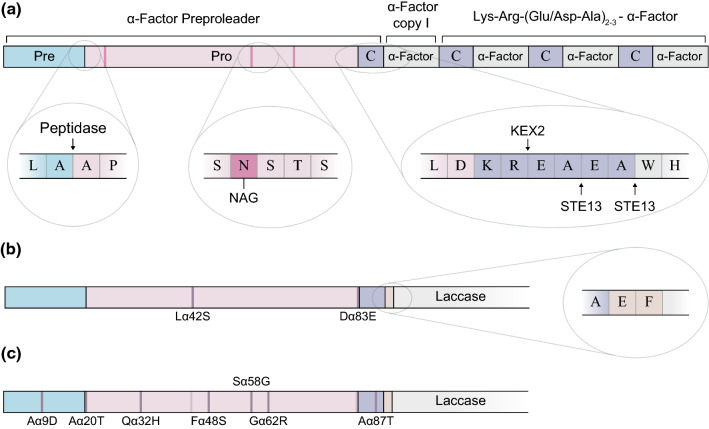
Scheme of MFα1 gene from *S. cerevisiae* and signal peptides. **a** The α-factor preproleader consists of a pre-region, a pro-region (with three *N*-glycosylation sites) and the first spacer (with KEX2 and STE13 cleavage sites). The signal peptide is followed by four α-factor gene copies separated by spacers (C) of different length. **b** The α_nat_ leader used in this study containing Lα42S and Dα83E mutations from pPICZα (Invitrogen) and the extra Glu-Phe residues after the spacer (light orange). **c** Evolved α_9H2_ leader with Aα9D, Aα20T, Qα32H, Fα48S, Sα58G, Gα62R, Aα87T mutations (dark purple), as well as Lα42S, Dα83E mutations (light purple) and extra Glu-Phe (light orange)

MFα1 gene structure consists of a signal sequence, known as α-factor preproleader, fused to four copies of the 13-residue α-factor protein, each of them preceded by a spacer peptide of 6–8 amino acids [Lys-Arg-(Glu/Asp-Ala)_2–3_] (Fig. 1a) [15–17]. By contrast to short canonical signal peptides (20–30 aa) [10], the α-factor preproleader comprises a long sequence of 89 amino acids divided into three structural parts: a 19-residue pre-region, a 64-residue pro-region and the first spacer of 6 amino acid residues. The pre-region displays a typical signal peptide structure with the following common motifs: a N-terminal positive charged domain, an hydrophobic core, and a final polar C-terminal end followed by the 64 residues of the pro-region. The latter and the spacer drive the final processing and release the α-factor mating pheromone to the extracellular media [16–18]. Moreover, the pro-region has three *N*-glycosylation sites that, although not being essential for secretion, appear to facilitate the transport from Endoplasmic Reticulum (ER) to Golgi apparatus [19, 20].

During their processing in *S. cerevisiae*, proteins directed by the α-factor preproleader are believed to be translocated across the ER membrane to the lumen in a post-translational pathway [21, 22]. Then, the pre-region is cleaved by an undetermined signal peptidase between the 19th and 20th positions [17, 23, 24], and the addition of *N*-linked glycosylation in the pro-region occurs [19]. At this point, the pro-protein is driven to its final post-translational processing at Golgi apparatus [25, 26]. Once there, KEX2 protease processes the peptide behind the dibasic sequence Lys84-Arg85 [17, 27], remaining four extra amino acids at the N-terminus of the protein. After their final cleavage by the action of the STE13 protease, the mature protein form is released to the culture media [28]. Although the yeast can manage the secretion of enzyme with the pre-region alone, the pro-region is important to facilitate the secretion [29]. It is generally assumed that the pro-region provides a proper transit of nascent peptides from ER lumen to Golgi apparatus in a COPII dependent pathway [22, 25, 26, 30, 31]. However, some studies point its role at protein translocation to the ER lumen [12, 13].

The versatility of the α-factor preproleader has been utilised for functional expression in yeast of a wide range of proteins such as fungal proteins [12, 32–34], green fluorescence proteins (GFPs) [13] or vaccines and pharmaceutical products (e. g. human insulin) [14]. One step further is the engineering of the signal peptide to raise the protein levels. Random mutagenesis of the α-factor preproleader improved Interleukin secretion more than twofold and highlighted the importance of increased hydrophobicity from 63rd to 66th positions of the pro-region [20]. In this work line, mutations accumulated in the α-factor preproleader during the directed evolution of fungal laccases fused to this signal sequence improved laccase secretion levels up to 40-fold [32] while demonstrated the crucial role of mutations at the pre-region for enzyme secretion [32, 33]. Other rational studies revealed the importance of single mutations such as Lα42S [35] or the role of specific motifs in the pro-region [25].

Laccases (EC 1.10.3.2) are multicopper oxidases able to oxidize phenols, aromatic amines, N-heterocycles, thiols and some metals. In fungi, they play a crucial role in wood delignification, and are involved in other processes such as detoxification, morphogenesis, pathogenesis and response to stress [36, 37]. Four copper ions participate in the catalysis; the monovalent oxidation of the substrate occurs at the T1 copper site; then, four electrons are transferred to the tri-nuclear cluster, formed by one T2 and two T3 copper ions, where oxygen is reduced to water [38, 39]. The high redox potential at T1 copper of certain basidiomycete laccases (around + 800 mV), high stability and substrate versatility, together with the use of oxygen from the air as sole requirement and the release of water as only by-product, make them green biocatalysts of choice for different industrial sectors [40]. Nevertheless, most wild basidiomycete strains produce low laccase levels and laccase heterologous expression is difficult, being a suitable target for directed evolution [32, 33, 41].

Several mutated α-factor preproleader sequences with improved secretory properties have been reported so far [20, 32–35, 42]. In particular, the co-evolution of this signal peptide fused to different fungal laccases during enzyme-directed evolution campaigns carried out in *S. cerevisiae* successfully enhanced laccase secretion [32, 33, 42–44]. However, designing an improved “universal” signal peptide capable of enhancing yeast production of a variety of different enzymes remains as a challenging task. We describe here a dual engineering approach of the α-factor preproleader to increase its ability to secrete recombinant enzymes and to add insights into its structure. We conducted a bottom-up optimisation design based on the mutations accumulated in α_9H2_, a recently evolved α-factor preproleader that contributes to the highest yields reported so far for a basidiomycete laccase produced in *S. cerevisiae* [42], and on other mutations selected in previous directed evolution campaigns, to study their influence alone as well as the interactions between them. In parallel, a top-down design served us to eliminate possible deleterious mutations accumulated in α_9H2_ leader. The secretory potentials of the α-factor leader sequences derived from both pathways were tested with two fungal laccases sharing ~ 60% sequence identity: the engineered PK2 laccase (Polyporales origin), obtained in the same evolution campaign than α_9H2_ [42], and a laccase synthesised de novo from the Agaricales fungus *Agrocybe pediades* (ApL, unpublished data). The optimised signal peptide was subsequently evaluated with other fungal oxidoreductases and hydrolases to asses its ability as an all-purpose leader to improve the secretion of different types of enzymes by the yeast.

## Results

In a previous work, we proved the capability of the evolved α_9H2_ leader [42] to improve the secretion by *S. cerevisiae* of diverse laccases compared to other evolved signal peptides [46]. The α_9H2_ leader differs from the native α-factor preproleader, α_nat_ leader from now on (Fig. 1b, Fig S1), in seven mutations (Fig. 1c) accumulated through subsequent evolution campaigns. Mutations Aα9D, Fα48S, Sα58G, Gα62R were added during the directed evolution of *Pycnoporus cinnabarinus* laccase (PcL) for functional expression in *S. cerevisiae* [32], and Aα87T during the evolution of PM1 laccase (PM1L [33]); all accumulated in the leader sequence of 7D5 chimeric laccase after DNA shuffling of evolved PcL and PM1L [44]. The Aα20T and Qα32H mutations were selected during subsequent evolution of 7D5 laccase to obtain PK2 variant [42]. It is worth noting that α_nat_ and α_9H2_ leaders contain 2 extra mutations (Lα42S and Dα83E) with respect to the original MFα1 gene [18]. Both mutations come from the α-factor preproleader of Invitrogen (inserted in pPICZα plasmids [35]). In addition, the α_nat_ leader we used here holds a *Eco*RI restriction site that was introduced to facilitate genetic engineering and encodes for a Glu-Phe extra sequence downstream the spacer and before the foreign protein (Fig. 1b, c).

First, the secretory potentials of the α_9H2_ leader was compared with the α_nat_ leader for laccase production. Both signal sequences, fused to the CDS of PK2 and ApL laccases were cloned in the pJRoC30 expression vector to transform *S. cerevisiae* cells. Yeast clones were grown in 96-well plates in SEM laccase expression medium [45] and the secreted activity was determined by the oxidation of ABTS (absorbance peak at 418 nm). While both construction gave detectable laccase activity, α_9H2_ leader provided significantly higher laccase activity levels than α_nat_ leader, roughly twofold for PK2 and 12-fold for ApL (Fig. S2), confirming previous results obtained with ApL [46]. Due the superiority of α_9H2_, it was used as upper reference leader in this study. Two engineering strategies were carried out: (i) a bottom-up process over α_nat_ to study the individual effect of mutations accumulated in α_9H2_ and others, and their epistatic interactions, and (ii) a top-down process over α_9H2_ to get rid of possible deleterious mutations accumulated during the in vitro evolution pathway that could mask the effect of real beneficial mutations.

### Bottom-up design of α_nat_ leader

**Fig. 2:**
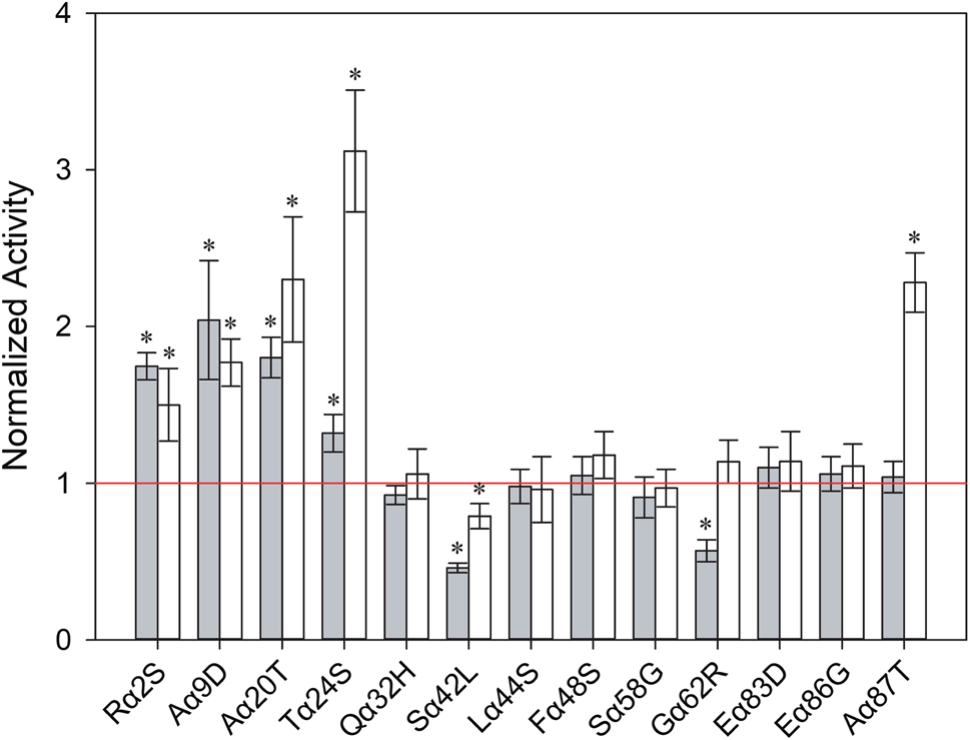
Laccase activities detected in *S. cerevisiae* microcultures expressing either PK2 (grey bars) or ApL (white bars) fused to the different single-mutated α leaders. Laccase activities were normalized to that of the corresponding parent type α_nat_-PK2 or α_nat_-ApL (red line). Error bars correspond to the error propagation of ten replicates of each parent type or individual mutant. Asterisks highlight significant differences between individual mutants and parent types according to Tukey's range test (95% confidence)

Site-directed mutagenesis on α_nat_ leader was performed to independently assess the effect of the following mutations: (i) the seven mutations accumulated in α_9H2_ leader (Aα9D, Aα20T, Qα32H, Fα48S, Sα58G, Gα62R, Aα87T) that were individually added to α_nat_ leader, (ii) the two mutations found in α_nat_ from pPICZα plasmid (Lα42S, Dα83E) that were removed individually, and (iii) four potentially beneficial mutations (Rα2S, Tα24S, Lα44S and Eα86G) selected in previous studies [32, 33] that were added individually to α_nat_ leader. The resulting 13 single-mutated α_nat_ leaders were fused to each laccase CDS (PK2 and ApL), cloned and expressed in *S. cerevisiae*. Laccase activities secreted by ten replicates of each clone grown in 96-well plates were screened with ABTS as substrate. The average laccase activity of each single mutant was normalized to the parental activity (with α_nat_ leader), and similarities and differences in secretion were statistically supported by the Tukey's range test. Satisfactorily, most of the mutations showed the same behaviour for the production of both laccases (Fig. 2). Clearly beneficial mutations were localised in the pre-region or near it; Rα2S, Aα9D and Aα20T augmented around twofold the secretion of both laccases, whereas Tα24S mutation improved their secretion dissimilarly (1.3-fold for PK2 and threefold for ApL). On the other hand, Qα32H, Lα44S, Fα48S, Sα58G had no effect on the secretion of PK2 and ApL, while Gα62R mutation had a detrimental effect on PK2 secretion (0.57-fold) and neutral effect on ApL. Mutations located in the spacer region had different effects: Eα86G seemed to have no influence on laccase secretion, while Aα87T highly improved the activity levels of ApL (2.3-fold) but not of PK2. Removal of Lα42S mutation decreased laccase secretion to 0.8-fold (ApL) and 0.4-fold (PK2) the activities detected with α_nat_ leader. Reversion of Dα83E had no effect. Nevertheless, both mutations, beneficial Lα42S and neutral Dα83E, were maintained in next assays because they were originally present in α_nat_ leader from Invitrogen and every substitution selected during the evolution to α_9H2_ leader could have had epistatic interactions with them.

**Fig. 3:**
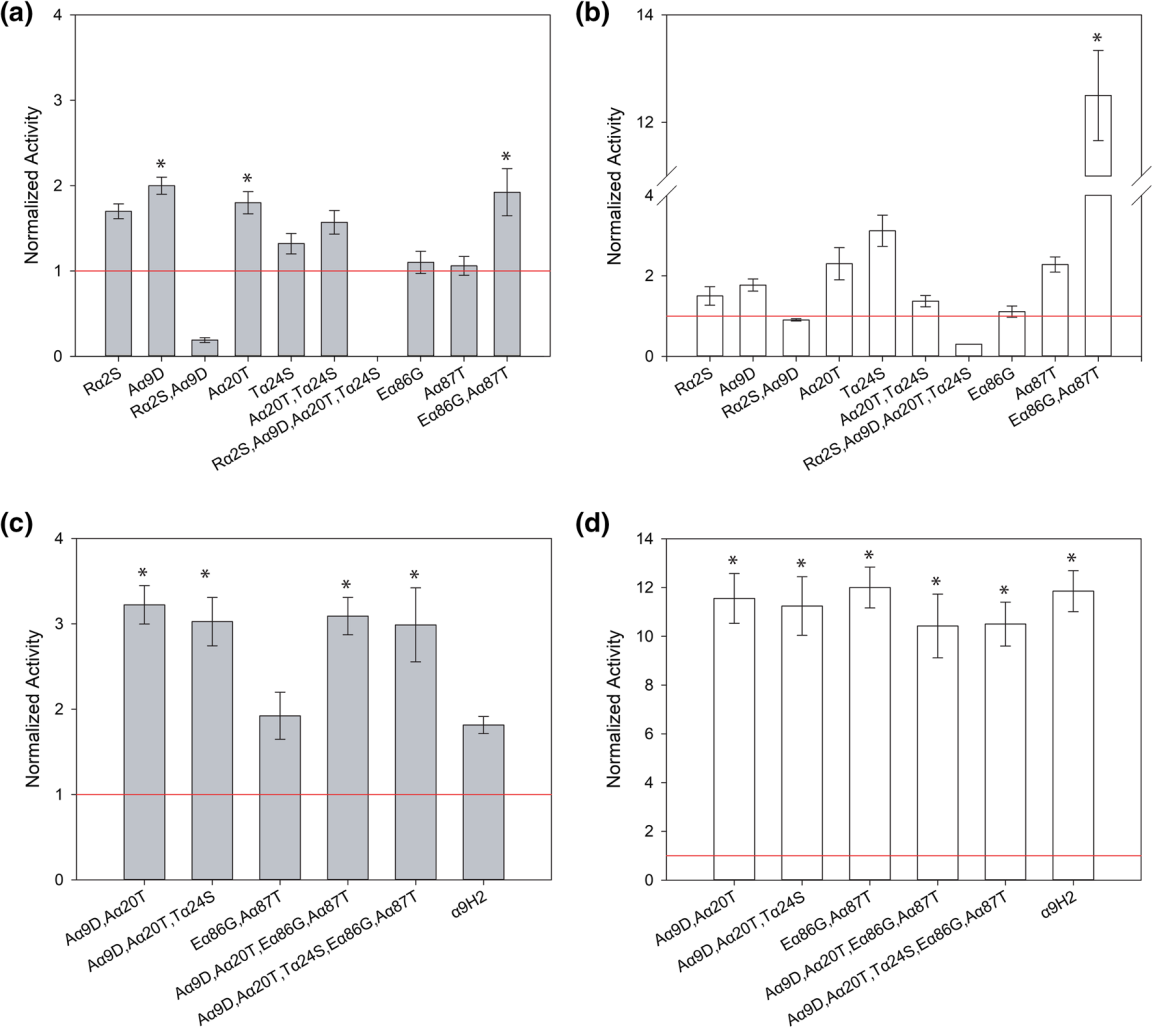
Laccase activities detected in *S. cerevisiae* microcultures expressing either PK2 (**a**, **c**) or ApL (**b**, **d**). fused to individual, double and quadruple α-preproleader mutants (**a**, **b**), or to the best mutated α leaders (α_A9D,A20T_ and α_A9D,A20T,T24S_) and the products of recombination with the second best (α_E86G,A87T_) (**c**, **d**). Secreted activities were normalized to those of the corresponding parent types: α_nat_-PK2 or α_nat_-ApL (red line). Error bars correspond to the error propagation of ten replicates of each parent type or mutant. Asterisks indicate the highest laccase activities according to Tukey's range test (95% confidence)

Next, we analysed the potential synergism between beneficial mutations Rα2S, Aα9D, Aα20T and Tα24S. Based on a proximity criteria, double (α_R2S,A9D_ and α_A20T,T24S_) and quadruple (α_R2S,A9D,A20T,T24S_) mutants of α_nat_ leader were obtained and fused to ApL and PK2 laccases. In addition, we built a double mutant (α_E86G,A87T_) at the spacer region. Again, ten replicates of each *S. cerevisiae* clone expressing the aforementioned constructions were grown in 96-well plates; the activities of the supernatants were measured and the corresponding average activities normalized to the laccase activity obtained with the α_nat_ leader (Fig. 3a, b). All data were supported by Tukey´s range test. The α_R2S,A9D_ leader diminished laccase secretion with respect to α_nat_ leader (to 0.2-fold for PK2 and 0.9-fold for ApL). The α_A20T,T24S_ leader favoured ApL secretion as compared to α_nat_ leader (1.4-fold), but the combination of both mutations was detrimental compared to the activity levels obtained with the single-mutated leaders α_A20T_ and α_T24S_. Conversely, the use of α_A20T,T24S_ leader with PK2 resulted in similar improvement than that obtained with α_T24S_. The quadruple mutant α_R2S,A9D,A20T,T24S_ led to minimal laccase levels (not detectable with PK2 and 0.3-fold with ApL). Surprisingly, α_E86G,A87T_ notably enhanced production of both laccases, around twofold for PK2 and 12-fold for ApL, suggesting a positive epistatic effect between both mutations.

Afterwards, to allow exploration of other possible advantageous combinations between Rα2S, Aα9D, Aα20T, Tα24S, Eα86G and Aα87T mutations, the 6 individual mutants, 3 double and 1 quadruple mutated α-factor leaders were subjected to in vivo recombination in *S. cerevisiae,* using PK2 as model laccase (Fig. S3a). After screening 1600 clones of the library, laccase activities were normalized to the activity obtained with the α_nat_ leader (Fig. S3b). Besides, α_E86G,A87T_ leader was included in the comparison as upper reference because it had produced one of the highest total activity improvements with PK2 (and the highest for ApL, Fig. 3a, b)*.* The five fittest clones carried α_A9D,A20T,T24S_ and α_A9D,A20T_ leaders. Mutation Rα2S was discarded for future assays since it seemed to be incompatible with the others (Fig. 3a, b).

Finally, since the combination of the winner set of mutations α_A9D,A20T_ and α_A9D,A20T,T24S_ with one of the best (α_E86G,A87T_) have not been selected from the in vivo DNA recombination assay, we synthesised two final leaders: α_A9D,A20T,T24S,E86G,A87T_ and α_A9D,A20T,E86G,A87T_ to evaluate their joint effect. It was confirmed that α_A9D,A20T,T24S_ and α_A9D,A20T_ leaders significantly raised laccase secretion with respect to α_nat_ (Fig. 3c, d), being the increment more pronounced with ApL (10–12-fold) than with PK2 (threefold). Conversely, based on Tukey´s range test, the production of none of the two laccases tested was improved by the addition of Eα86G and Aα87T mutations to these leaders. We therefore discarded the latter mutations from the final optimised signal peptide. Finally, it was evidenced the neutral effect of Tα24S mutation, so it was discarded as well. In conclusion, we selected α_A9D,A20T_ as the optimised leader from the bottom-up process, because it remarkably surpassed the secretion potential of α_nat_ leader to values similar (12-fold for ApL) or better (threefold for PK2) than those obtained with α_9H2_ leader.

### Top-down design of α_9H2_ leader

**Fig. 4:**
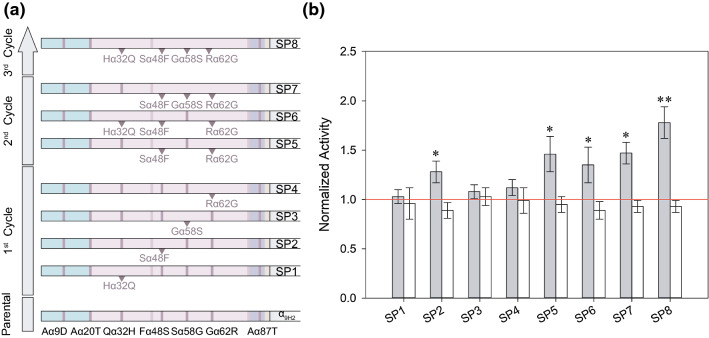
Top-down strategy over α_9H2_ leader. **a** Scheme summarizing the three cycles of top-down design of α_9H2_ leader directed to improve laccase secretion by removing possible non-beneficial mutations. The removed mutations are highlighted in each α leader sequence (SP1-SP8); colour codes correspond to those shown in Figure 1. **b** Laccase activities detected in *S. cerevisiae* microcultures expressing either PK2 (grey bars) or ApL (white bars) fused to the different reverted α_9H2_ mutants (SP1-SP8). Laccase activities were normalized to that of the corresponding parent type α_nat_-PK2 or α_nat_-ApL (red line). Error bars correspond to the error propagation of ten replicates of each parent type or mutant. One asterisk indicates significant differences respecting the parent type and two asterisks highlight the clone with significant highest activity among all, according to Tukey's range test (95% confidence)

In the top-down approach we aimed to obtain an optimised and simplified version of the α_9H2_ leader by removing possible deleterious or neutral mutations that could have been introduced during its in vitro evolution pathway and might mask the effect of beneficial mutations accumulated in the signal peptide. In a first cycle, mutations Qα32H, Fα48S, Sα58G and Gα62R were individually reverted from the α_9H2_ leader since their neutral effect on laccase secretion were confirmed during the bottom-up approach. The resultant α-factor leaders (Signal Peptides) were named as follows: SP1 (Hα32Q mutation), SP2 (Sα48F mutation), SP3 (Gα58S mutation) and SP4 (Rα62G mutation) (Fig. 4a). SP1, SP3 and SP4 had no significant effect on the secretion of PK2 or ApL, as compared to the laccase activities detected with α_9H2_ leader. SP2 did not improved ApL production, but raised 1.3-fold the production of PK2, suggesting a possible deleterious effect of Fα48S mutation in α_9H2_ leader for secretion of this laccase (Fig. 4b).

In a second evolution cycle, given the aforementioned detrimental effect of Fα48S mutation in α_9H2_ leader for PK2 laccase (Fig. 4b), and of Gα62R observed in the bottom-up approach also for PK2 (Fig. 2), both mutations were simultaneously reverted in SP5. It increased secretion of PK2 similarly to SP2 (with only reversion of Fα48S mutation), whereas no improvement in ApL levels were observed respecting α_9H2_. Two more leaders were designed in parallel to assess the effect of Qα32H and Sα58G mutations on SP5 environment; each had three reverting mutations: SP6 (Hα32Q, Sα48F, Rα62G) and SP7 (Sα48F, Gα58S, Rα62G). Alike SP5, SP6 and SP7 provided similar levels of PK2 than SP2, confirming the detrimental effect of Fα48S mutation on α_9H2_ leader for laccase secretion.

Lastly, the combined absence of the four mutations Qα32H, Fα48S, Sα58G and Gα62R was assayed in SP8 leader. SP8 showed no effect on ApL laccase secretion, whereas the simultaneous reversion of the four mutations rendered significant higher (1.8-fold) PK2 laccase levels as compared with α_9H2_ leader. Despite the fact that Fα48S seemed to be the only deleterious amino acid change in α_9H2_ leader for PK2 laccase secretion (SP2, Fig. 4b), the four aforementioned mutations have a larger deleterious effect together than separately. Thus, mutations Aα9D, Aα20T and Aα87T seem to be responsible for the greater secretory potential of α_9H2_ with respect to α_nat_ leader.

### Selection of a final optimised α-factor leader

**Fig. 5:**
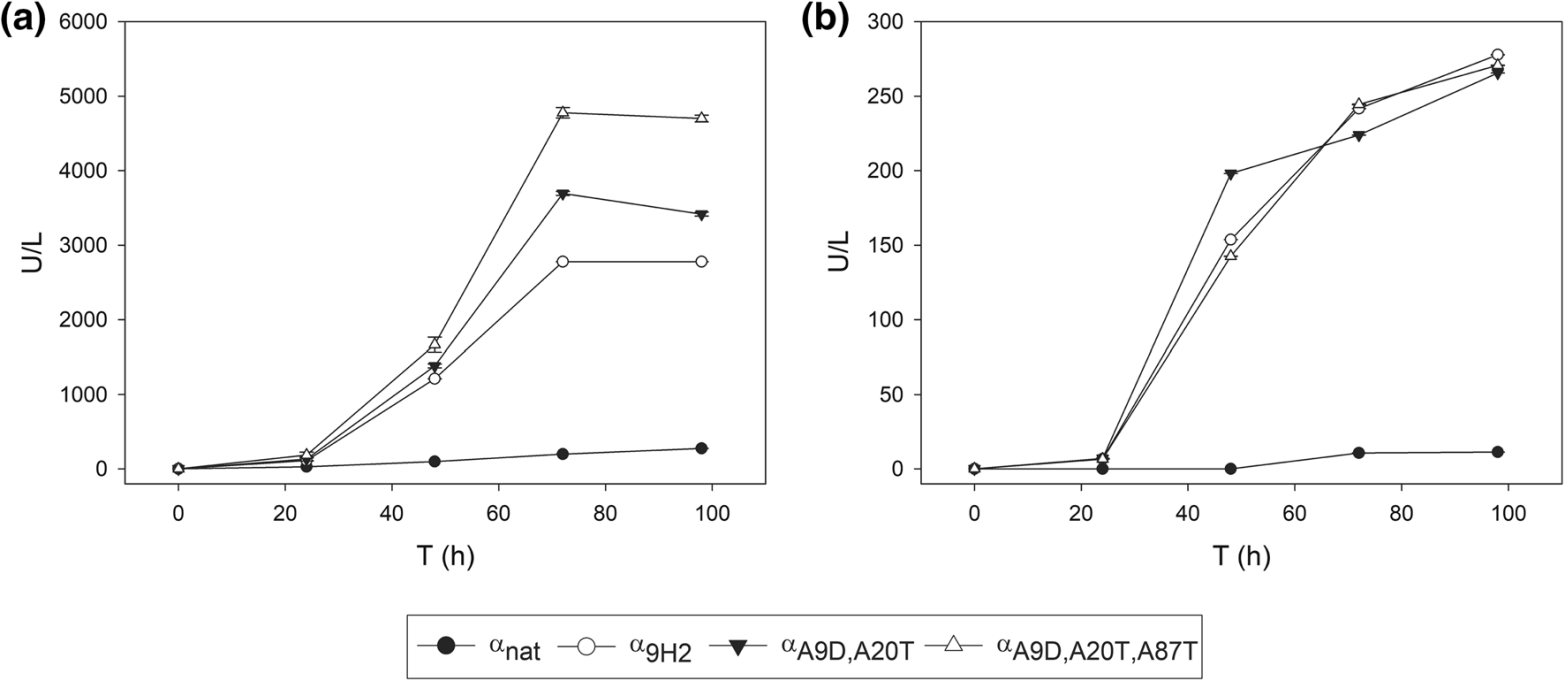
Flask production of PK2 (**a**) and ApL (**b**) laccases by *S. cerevisiae* with the best α-factor leaders obtained in the bottom-up (α_A9D,A20T_) and top-down (α_A9D,A20T,A87T_) designing strategies compared with α_nat_ and α_9H2_ leaders_._ Laccase activity (U/L) was measured with ABTS pH 3. Error bars indicate standard derivation of three flask replicates

The two final leaders selected from the bottom-up (α_A9D,A20T_) and top-down (α_A9D,A20T,A87T_) pathways were compared for secretion of ApL and PK2 laccase by *S. cerevisiae* cultured in flasks. The α_nat_ and α_9H2_ leaders were included in the assay as lower and upper references. In this case, the minimum laccase expression medium (SEM) utilised in the micro-fermentations was replaced by a richer medium (EB) because the reduced growth of the yeast in SEM could limit laccase production in flasks [45]. We aimed as well to check the reproducibility of the results obtained in other culture medium and conditions. Optical densities (Fig. S4) and laccase activities (Fig. 5) of the cultures were monitored for 4 days. All *S. cerevisiae* clones grew similarly, but they produced dissimilar laccase activities. After 4 days of incubation, α_A9D,A20T,A87T_ leader fused to PK2 laccase provided up to 4800 U/L with ABTS, while α_A9D,A20T_ yielded around 3700 U/L, which respectively represent 18-fold and 14-fold higher activities than that detected with α_nat_ leader, and 1.8-fold and 1.4-fold improvements respecting laccase levels detected with α_9H2_ leader (Fig. 5a). On the other hand, α_A9D,A20T,A87T_, α_A9D,A20T_ and α_9H2_ leaders fused to ApL gave rise to similar laccase levels (around 260 ABTS U/L), which represent a 26-fold improvement respecting the laccase activity detected with α_nat_ (Fig. 5b). The superior secretory potential of the three engineered leader sequences with respect to α_nat_ was therefore confirmed. Furthermore, the similar (ApL) or markedly improved (PK2) laccase levels obtained with the optimised leaders with respect to the α_9H2_ leader, pointed out the essential role that Aα9D and Aα20T mutations play in the superior secretory capability of α_9H2_ leader. By contrast, the dissimilar results obtained with α_A9D,A20T_ or α_A9D,A20T,A87T_ in the production of the two laccases suggested that the variable effect exerted by Aα87T mutation may be influenced by the sequence of the fused protein.

Taking all this into account, α_A9D,A20T_ was selected over α_A9D,A20T,A87T_ leader. Since α_A9D,A20T_ leader carried also mutations Lα42S and Dα83E (Invitrogen), we double checked their contribution in α_A9D,A20T_ leader context by individually discarding them from the selected leader fused to PK2 and ApL. As previously shown, the absence of Lα42S had a strong negative effect on laccase production (0.5-fold reduction), whereas we confirmed the neutral effect of Dα83E (Fig. S5). Mutations Aα9D, Aα20T, Lα42S, Dα83E were included in the optimised all-purpose leader for further assays, named α_OPT_ from now on.

### Expression of other enzymes

**Fig. 6:**
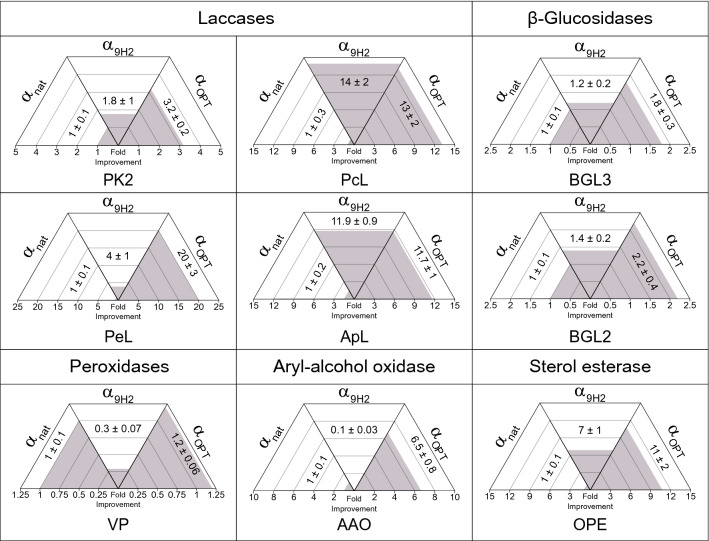
Enzyme production by *S. cerevisiae* cultured in 96-well plates using α_nat_, α_9H2_ or α_OPT_ leaders. Secreted enzymatic activities of laccases (PK2, ApL, PeL, and PcL), aryl-alcohol oxidase (AAO), peroxidase (VP), β-glucosidases (BGL2 and BGL3) and sterol esterase (OPE) are indicated as fold improvements with respect to the activities obtained with α_nat_ leader. Error correspond to the error propagation of ten replicates of each construction (with α_nat,_ α_9H2_ or α_OPT_)

The secretory potential of α_OPT_ leader was evaluated for the production by *S. cerevisiae* of other fungal oxidoreductases like two more laccases from *Pleurotus eryngii* (PeL) [46] and *Pycnoporus cinnabarinus* (PcL) [32], an aryl-alcohol oxidase from *P. eryngii* (AAO) [47] and a versatile peroxidase (VP) from *P. eyringii* [48]. Besides, we assayed it with fungal hydrolases such as two β-glycosidases (BGL2 and BGL3) from *Talaromyces amestolkiae* [49, 50] and a sterol esterase (OPE) from *Ophiostoma piceae* [51]. To this aim, the native signal peptides were removed and replaced by α_OPT_, α_nat_ and α_9H2_ leaders for enzyme expression in *S. cerevisiae* (the two latter used as lower and upper references). Yeast cells (ten replicates of each clone) were grown in 96-well plates in SEM, and the secreted enzyme activities were measured and normalized to the activities obtained with α_nat_ leader (Fig. 6).

In general, α_OPT_ leader provided enzyme secretion levels significantly higher than those obtained with α_nat_ leader. In some cases, the increments in enzyme production obtained with α_OPT_ leader were remarkable: 10–20-fold higher levels for PeL, PcL, ApL and OPE than those obtained with α_nat_ leader (Fig. 6). As regards α_9H2_ leader, it significantly enhanced the production of all tested laccases respecting the use of α_nat_ leader, but to a lower or at most similar extend than α_OPT_ leader. Moreover, α_9H2_ performance with the rest of enzymes was not as good, being in general similar or worse than α_nat_ leader (e.g. 0.35-fold for VP and 0.07-fold for AAO). Taking all this into account, α_OPT_ leader emerges as a general signal peptide suited for efficient expression of fungal enzymes in *S. cerevisiae*.

### Combinatorial saturation mutagenesis on the spacer region

**Fig. 7:**
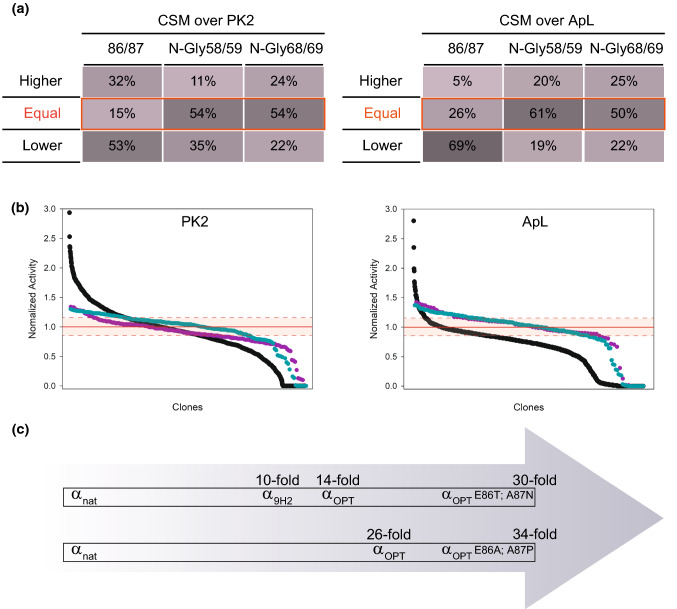
**a** Percentages of clones with higher, lower or parent-like activities of mutant libraries obtained upon mutation of positions 86th and 87th of the spacer region (black) and on the 2nd and 3rd positions of NXT/S sequence of the *N*-glycosylation sites 57 (purple) and 67 (cyan) of α_OPT_ leader for secretion of laccases PK2 and ApL (interval of Confidence of 95%). **b** Activity landscapes of the aforementioned CSM86/87, CSM-*N*Gly58/59 and CSM-*N*Gly68/69 mutant libraries of α_OPT_ leader fused to laccases PK2 or ApL. The activities of the clones are shown as relative to the laccase activities obtained with α_OPT_ leader (depicted as 1); the interval of confidence of the CSM86/87 assay is indicated with dashed lines. **c** Secretion improvements for PK2 (top) and ApL (bottom) obtained throughout α-factor preproleader engineering, from α_nat_ to α_OPT_ mutated in 86/87

Mutations Eα86G and Aα87T were ruled out from the aforementioned “universal” α_OPT_ leader due to their dissimilar effect on secretion of ApL or PK2 laccases which might be related to the different fused protein sequences. We hypothesised that positions 86th and 87th of the spacer region would play a crucial role in the secretory potential of the signal peptide, and, therefore, they may well be hotspots for engineering the α leader towards the production of a particular recombinant enzyme. To test this hypothesis, positions 86th and 87th of α_OPT_ leader fused either to PK2 or ApL were subjected to combinatorial saturation mutagenesis (CSM), covering all possible amino acid combinations, and the activities of the mutant libraries expressed in *S. cerevisiae* were screened with ABTS. Population of clones with parental-like activity (inside parent’s confidence interval) were minor in both CSM 86/87 libraries, whereas most clones (53% for PK2 and 69% for ApL) exhibited lower activity than parental α_OPT_ leader, and clones with higher laccase activities represent a 32% in PK2 library and 5% in ApL library (Fig. 7a).

On the other hand, we randomised positions 58/59 and 68/69 of two *N*-glycosylation sites (Asn in positions 57th and 67th) of the pro-region of the α leader [19, 52], in such a way that the consensus pattern Asn-X-Ser/Thr was conserved. While Asn was maintained, positions 58 and 68 were mutated by whatever amino acid except for Pro and positions 59 and 69 were restricted to Ser or Thr. We used the resulting CSM *N*-Gly58/59 and *N*-Gly68/69 libraries (built on α_OPT_-PK2 and α_OPT_-ApL) as reference of presumably neutral libraries, and compared the results from their screening with those obtained from the CSM86/87 libraries under the criteria “the larger population of clones with parental-like activity, the less impact the mutated sites have on enzyme secretion”. By contrast to CSM 86/87 libraries, most of the clones (50–60%) exhibited parental-like activities (Fig. 7a), confirming that the 2nd position of *N*-glycosylation sites was not so relevant for α leader engineering as 86th and 87th positions were. In addition, although clones with improved activities were also found in CSM *N*-Gly58/59 and 68/69 libraries, the improvements detected were significantly lower. Moreover, the plain shape of their activity landscapes remarks the “neutral” nature of these libraries by contrast with the hill trend of CSM86/87 landscapes (Fig. 7b).

The best amino acid substitutions selected from each CSM 86/87 library were different for PK2 laccase (Eα86T/Aα87N; Eα86D/Aα87N and Eα86D/Aα87G) and ApL (Eα86A/Aα87P; Eα86T/Aα87K and Eα86S/Aα87R). The clones providing the highest secreted activity improvements (α_OPT Eα86T/Aα87N_ for PK2 and α_OPT Eα86A/Aα87P_ for ApL) were cultivated in flask to test laccase production. Production of PK2 laccase was raised roughly twofold and 30-fold as compared with the activity levels provided by α_OPT_ leader and α_nat_ leader, respectively; while for ApL production, the improvements were around 1.3-fold and 34-fold, respectively (Fig. 7c).

Finally, we purified PK2 laccase produced with α_OPT_ and α_OPT Eα86T/Aα87N_ as leaders in *S. cerevisiae* flask cultures (Fig. S6). In both cases, after deglycosylation with Endo H, the enzyme showed a molecular weight around 53 KDa, coincident with its theoretical MW (Fig. S7). The enzymes purified from both cultures showed also identical specific activities with ABTS regardless of the signal peptide used: 405 ± 23 U/mg and 423 ± 34 U/mg for the enzyme secreted with α_OPT_ and α_OPT Eα86T/Aα87N_, respectively. With this data and the laccase activity units detected in the culture broths (2800 U/L with α_OPT_ and 4800 U/L with α_OPT Eα86T/Aα87N_), we determined that the total mg of PK2 laccase secreted with α_OPT Eα86T/Aα87N_ was roughly twice as high the amount of enzyme secreted with α_OPT_.

## Discussion

We present here the designing of an optimised version of the α-factor preproleader from *S. cerevisiae* to improve the production of fungal enzymes by the yeast. The α_9H2_ leader developed in our lab [42] was selected as reference signal peptide because this mutated leader significantly improves the secretion by *S. cerevisiae* of several laccases compared with other evolved α leaders obtained in our lab [46], or with the α_nat_ leader (Invitrogen) as shown here for PK2 and ApL. Thus, we studied the effect of the mutations accumulated in α_9H2_ leader sequence through its evolution pathway as well as other mutations of the α-factor preproleader selected (and eventually lost) during successive laccase directed evolution campaigns [32, 33, 42, 44]

Two engineering pathways of the signal peptide were carried out: a bottom-up designing strategy on α_nat_ leader and a top-down one on α_9H2_ leader using PK2 and ApL laccases as model enzymes. In total, 13 candidate mutations were assayed (alone or combined) until both approaches met to obtain the optimised leader α_OPT_. The superior secretory potential exhibited by α_OPT_ leader, as compared with α_nat_ or α_9H2_ leaders in different media and culture conditions, arises from the accumulation of four mutations, two beneficial mutations Aα9D, Aα20T from α_9H2_ leader, and Lα42S, Dα83E from α_nat_ leader (Invitrogen). Actually, the Lα42S mutation is clearly beneficial as demonstrated by the significantly diminished laccase secretion levels when it was reverted in α_nat_ and α_OPT_ leaders; whereas reversion of Dα83E mutation had a neutral effect. Despite this, we opted to maintain it as well, given it was also present in the original α_nat_ leader and it adds an *Xho*I cleavage site to facilitate further genetic engineering.

Mutation Lα42S is absent in the original α-factor preproleader sequence [15] as well as in the MFα1 of S288C strain, the first *S. cerevisiae* genome released in 1996 [53, 54]. Moreover, mutations Lα42S, Dα83E are not found in none of the MFα1 sequences from *S. cerevisiae* strains available in Saccharomyces Genome Database (SGD; https://www.yeastgenome.org) (Fig. S8). Interestingly, Lα42S mutation come out when MFα1 *S. cerevisiae* gene was first simultaneously sequenced in two works [15, 16]. Both articles published in agreement the same tandem gen structure sequence except for 42nd residue, which was a Ser in Kurjan and Herskowitz [16] instead of the Leu found in the sequence published by Singh and co-workers [15] and in the rest of MFα1 sequences published afterwards. From a population of 50,000 transformed cells with YEp13 plasmid containing the expected MFα1 gene, Kurjan and Herskowitz selected a possible α-mating factor overproducer colony based on morphology criteria. Thus, and according to our own results, it is most probable that Lα42S mutation comes from a random mutational event and it would have conferred a dominant phenotype to this single colony, favouring its selection [16].

As evidenced by our results, most mutations entailing a favourable effect on the heterologous production of both laccases were located at or near the pre-region (except for mutation Lα42S located in the pro-region). Single mutations Aα9D and Aα20T, from α_9H2_ leader, and Rα2S and Tα24S recovered from PcL directed evolution pathway [32], were proved to be beneficial during the bottom-up pathway. Interestingly, the beneficial effect of Rα2S confronts the commonly assumed requirement for the presence of positive charged residues at the amino terminal of signal peptides [10]. In this line, mutation Rα2F had been reported to have a neutral effect on somatostatin production, whereas the substitution of the third residue by a positive charged amino acid seriously attenuated the translocation across the ER membrane [55]. These results highlight the influence of first residues of the α-factor preproleader, although positively charged amino acids might not be mandatory. The latter seems to be more crucial in bacterial signal peptides, since in Eukaryotes the terminal Met is unformulated and remains positively charged, which seems to be enough to ensure the proper operation of eukaryotic signal peptides [56]. Nevertheless, the counteracting effect of Rα2S and Aα9D mutations put together (Fig. 3a, b), supported by the absence of the Rα2S and Aα9D combination in the fittest α-factor leader variants selected from the recombination library (Fig. S3), discarded Rα2S mutation for the final α_OPT_. This detrimental effect of Rα2S, Aα9D combination had been suggested during PcL directed evolution, where both mutations were selected separately but never simultaneously during the screening of DNA recombination libraries [32].

Mutation Aα9D provided remarkable improvements on enzyme secretion, regardless of the laccase attached to the signal peptide and in cooperation with Aα20T. Beneficial mutations in the hydrophobic core of the pre-region of the α-factor leader had been selected during the directed evolution of fungal laccases for functional expression in *S. cerevisiae*. Mutation Vα10D was selected during the directed evolution of PM1L where it notably raised the laccase activity detected in the supernatants [33]. In parallel, Aα9D stood out as the mutation of the α-factor preproleader responsible for the highest laccase improvement during PcL directed evolution [32]. Moreover, other mutation reducing the hydrophobicity of these positions (Vα10A) had been selected during the design of α-factor preproleader for antibody expression in *S. cerevisiae* [20]. Despite the hydrophobic core has been described to facilitate a proper translocation of the peptide into the endoplasmic reticulum [12, 57, 58], a shift towards hydrophilicity seems to be the only common element in the aforementioned amino acid substitutions (Aα9D, Vα10D, Vα10A). This trend was also observed in the α-factor preproleader from YJM339 *S. cerevisiae* strain (available at SGD) which held mutation Aα9T [59] (Fig. S8).

Mutation Aα20T produced good results similar to those obtained with Aα9D. The role of the former mutation seems to underlie in its location immediately before the cleavage site, between pre and pro-regions. In concordance with the consensus classical signal peptide structure, the pre-region conserves the AXA motif at the -1 and -3 positions relative to the cleavage site (Ala17-Leu18-Ala19) [24, 60]. Aα20T is likely to increase the efficiency of protease cleavage which is expected to be a limiting step in the secretion of some proteins [61]. Even though Tα24S mutation alone has a favourable effect on laccase secretion similar to that of Aα20T, no positive synergism between both mutations could be found (Fig. 3a, b). This added to its negligible effect on α_OPT_ leader context (Fig. 3c, d) led us to discard Tα24S. Mutations Tα24S and Sα58G are in first and second *N*-glycosylation sites of the signal peptide, specifically in the second position of the *N*-Gly consensus sequence (N-X-T/S). Replacement of this variable second residue may alter the affinity for sugar anchoring [62, 63]. However, in view of the results obtained from the bottom-up and top-down designing strategies with Sα58G mutation (Figs. 2 and 4), and the absence of relevant improvements on secretion of PK2 and ApL laccases in the mutants selected from CSM (*N*-Gly58/59 and *N*-Gly68/69 libraries), contribution of the second position of *N*-glycosylation sites to the secretory capability of the α-factor preproleader seems to be insignificant in comparison with other amino acid substitutions shown here.

Most of the studied mutations of the pro-region_,_ Qα32H, Lα44S, Fα48S, Sα58G, Gα62R, Dα83E, exhibited either neutral or deleterious effects on enzyme production, depending on the attached laccase. In this line, the larger detrimental effect of Qα32H, Fα48S, Sα58G, Gα62R mutations put together, for PK2 secretion, pointed out the relevance of the strategy we followed to detect negative epistasis among mutations. Only Lα42S mutation positively contributed to ApL and PK2 secretion, while Dα83E mutation (both coming from the original α_nat_ leader of Invitrogen) resulted neutral, in agreement with the effects observed for both mutations during GFP expression in *Pichia pastoris* [35]. The contribution of the pro-region to the overall function of the α-factor preproleader had been proved through deletion of the entire pro-region, which severely reduces the processing of the foreign enzyme in *S. cerevisiae* [64]. It has been also concluded that certain consecutive residues seem to ensure the proper functionality of the pro-region [12, 20]. The main disagreement lies on its precise function, either facilitating translocation across the ER lumen [12, 13], or acting as an ER exporting signal in a COPII vesicle-dependent way by means of the Erv29 protein recognition in *S. cerevisiae* [22, 25, 26]. Even when a definitive statement about the pro-region function cannot be given, all of the above points out the importance of some particular residues of this region. More specifically, mutation in the 42th position recurrently appears in the literature, supporting our results [20, 25, 35]. The potential of 42th and adjacent positions were reported in 2016 in the WO2015128507 patent [65], which contained a method for recombinant expression of a glucagon-like peptide-1 using α-factor preproleader variants bearing substitutions at 38–42 residues, including mutation Lα42S.

Shortcomings during KEX2 processing, derived in either over-saturation or inefficient cut, constitute a bottleneck in foreign protein secretion [66, 67]. STE13 protease seems not to be as crucial as KEX2, since several proteins retaining an extra N-terminus amino acid tail related to inefficiency of protease processing resulted in overexpression of the recombinant protein [34, 44, 68, 69]. Optimizing KEX2 cleavage site or integrating additional constitutively expressing KEX2 emerged as possible strategies to remove the aforementioned bottle neck [67]. However, the optimization of the cleavage site seems difficult given its fixed dibasic Lys84-Arg85 sequence that does not accept any substitution apart from Rα85K [67, 70, 71]. On the other hand, there are evidences about the importance of residues downstream the KEX2 cleavage site and before the mature protein [66, 72, 73]. This spacer region is variable in length and shows negatively charged amino acids in the four tandem genes [Lys-Arg-(Glu/Asp-Ala)_2–3_] of MFα1 gene, which shines light on its possible optimization.

Consistent with the above data, mutations Eα86G, Aα87T of the spacer region increased protein secretion yields, although with dissimilar results for ApL and PK2 laccases. However, their negligible positive effect in α_OPT_ context led us to discard them from the final optimised signal peptide. We hypothesised that the enzyme secretion could be significantly raised by randomising 86th and 87th positions of the α_OPT_ leader, being the amino acid substitutions most likely selected in the context of the fused protein. The contribution of these positions to tune the secretory potential of α_OPT_ was assessed through CSM of both positions, exploring the secretion of ApL and PK2 laccases. The CSM 86/87 libraries were compared with CSM *N*-Gly58/59 and *N*-Gly68/69 libraries designed in such a way that the *N*-glycosylation pattern required for pro-peptide processing [19, 52] was preserved. The activity landscapes of the CSM *N*-Gly libraries showed a clear predominance of clones with parental-like activities, confirming they were “neutral” libraries. By contrast, the low number of clones with parental-like activities found in CSM 86/87 libraries underlined the higher evolvability of positions 86/87. First, the significant laccase activity improvements found in a percentage of clones of both CSM 86/87 libraries evidenced that positions 86/87 constitute hotspots for engineering the α leader to improve enzyme secretion. Second, selection of different fittest 86/87 amino acid pairs for improving the secretion of PK2 or ApL, supported our hypothesis that these positions have to be optimised specifically for a given protein. In fact, the modest number of clones with improved activities found in CSM 86/87 library for ApL (5%) can be attributed to the presence of an already suited amino acid pair in α_OPT_ leader for the secretion of this laccase in particular. This is in agreement with results from saturation mutagenesis on 86th position of the α-factor preproleader that depended on the protein attached [67].

On the other hand, the purification and characterisation of PK2 laccase produced with α_OPT_ or α_OPT Eα86T/Aα87N_ as signal peptides, allowed us to confirm that the enzyme had been equally processed and it has the same catalytic activity, regardless of whether positions 86/87 of the signal peptide had been optimised or not. After deglycosylation with Endo H, the enzyme showed a MW coincident with its theoretical MW, indicating the cleavage of the pro-region by KEX2 in both signal peptides. Also, the equal catalytic activity of the enzyme produced with α_OPT_ or α_OPT Eα86T/Aα87N_ confirmed that the higher laccase activity detected in the supernatant of yeast culture producing α_OPT Eα86T/Aα87N_-PK2 were due to a higher level of secreted protein (roughly twice the amount produced with α_OPT_ leader). These results corroborate the contribution of the residues of the spacer region (after Arg85) for the correct processing of the pro-region of α-factor preproleader by KEX2 and, consequently, their influence in the overall enzyme secretion process [66, 67, 72, 73].

Finally, the “universal” optimised leader, α_OPT_, exhibited superior secretory potential with other fungal enzymes from different sources: basidiomycete oxidoreductases (versatile peroxidase, aryl-alcohol oxidase and two more laccases) and ascomycete hydrolases (two β-glucosidases and a sterol esterase). In general, α_OPT_ leader enhanced enzyme levels from roughly 2 to 20-fold those obtained with α_nat_, and also outperformed α_9H2_ for secretion of most enzymes tested, except for two cases (ApL and PcL) where similar values were obtained. Special mention should be given to the production of BGL2, BGL3 and OPE enzymes (96-well plate format), since this work constitutes the first report for functional expression and secretion of these enzymes by *S. cerevisiae*. On the other hand, even though PK2 [42], PcL [32], VP [34] and AAO [74] had been already expressed in the yeast, the production levels were enhanced using the signal peptide optimised here. Moreover, α_OPT_ leader showed similar behaviour in different media (SEM or EB) and culture conditions (microtiter plates or flask). Even though the differences between α_nat_ and α_OPT_ leaders are larger in richer medium during flask production, α_OPT_ leader maintains its superior secretory capability with ApL and PK2 regardless of the conditions used for yeast growth and laccase production, by contrast to reported medium-dependence of other evolved α-factor leaders for laccase expression [45].

## Concluding remarks

We present here an optimised version of the α-factor preproleader (α_OPT_ leader) obtained through a dual (bottom-up and top-down) designing strategy of the signal peptide. The systematic scrutiny and combination of mutations selected in previous enzyme-directed evolution campaigns allowed us to disclose the important role that particular regions of the α-factor preproleader, such as the pre-region or the spacer region, play in its functionality. The α_OPT_ leader is able to markedly enhance the secretion of a wide range of fungal enzymes in yeast as compared with the native α-factor preproleader (or with other mutated α-leaders). Additionally, we propose a guideline to further boost the production yields of a specific recombinant enzyme, through simultaneous randomisation of positions 86th and 87th of the spacer region of α_OPT_ fused to the target protein, followed by high-throughput screening of the CSM library to select the best mutants.

## Materials and methods

### Reagents and strains

Yeast Transformation Kit, *p*-nitrophenyl butyrate and *p*-nitrophenyl β-d-galactopyranoside, High Pure Plasmid Isolation Kit, ABTS (2,2′azinobis (3ethylbenzothiazoline- 6 sulphonic acid)), *p*-methoxybenzyl alcohol, and Horseradish peroxidise (HRP) were purchased from Merck. Restriction enzymes *Not*I and *Bam*HI were obtained from New England Biolabs. Phusion High-Fidelity DNA polymerase was obtained from NEB and QIAquick gel extraction kit from QIAGEN. Zymoprep™ Yeast Plasmid Miniprep II was purchased from Zymo Research. *S. cerevisiae* BJ5465 strain was purchased from LGC Promochem (Barcelona, Spain).

### Culture and media

Minimal Medium (MM) and EB expression medium were synthesised as it is described in Camarero and Co-workers [32]. SEM expression medium was synthesised as it was described in Mateljak and Co-workers [45], without including alcohol. Additionally, 4 mM and 2 mM CuSO_4_ were added for laccase expression in EB and SEM mediums, respectively. No cofactors were required for the rest of enzymes described in this study.

### Enzyme engineering in *S. cerevisiae*

I. *Agrocybe pediades* laccase [46], *Pleurotus eryngii* laccase (with two mutations to facilitate its functional expression) [46]. PK2 laccase [42], *Pycnoporus cinnabarinus* laccase [32], *P. eryngii* versatil peroxidase [48], *P. eryngii* aryl-alcohol oxidase [47], *Talaromyces amestolkiae* β-glucosidases [49, 50] and *Ophiostoma piceae* sterol esterase [51] were obtained from our collection of enzymes at CIB. The enzymes’ CDS were cloned in the uracil-independent and ampicillin resistant vector pJRoC30 with the α-factor preproleader from Invitrogen by In Vivo Overlap Extension (IVOE) [75]. The primers sense and antisense used are described in the supplementary material. The mutated α-factor leaders under study were also cloned in the pJRoC30 vector by IVOE. A first fragment was obtained by PCR with ExtFw sense primer and 87 Final-Rv antisense for α_9H2_ leader or NatFinal-Rv antisense for α_A9D,A20T_ or α_nat_ leaders, and the second fragment was obtained by PCR with 87Final-Fw sense for α_9H2_ or NatFinal-Fw sense for α_A9D,A20T_ or α_nat_ leaders and ExtRv anti-sense primer (Tables S1 and S2). The pJRoC30 was linearized with *Not*I and *Bam*HI restriction enzymes and transformed with the two PCR fragments in *S. cerevisiae* by IVOE.
II. Bottom-up design of α-factor leader. The single mutations Rα2S, Aα9D, Aα20T, Tα24S, Qα32H, Lα42S, Lα44S, Fα48S, Sα58G, Gα62R, Dα83E, Eα86G, Aα87T were incorporated in the sequence of the α-factor preproleader from Invitrogen by site-directed mutagenesis. The same PCR strategy previously described was used adding the suitable sense and anti-sense primers (Table S1). Double, triple and quadruple variants were obtained using a step-by-step addition of mutations.
III. Top-down design of α-factor leader. The Hα32Q, Sα48F, Gα58S, Rα62G single, double, triple and quadruple reverted mutants from α_9H2_ leader were obtained as described above.
IV. Recombinant library of α-factor preproleader mutants fused to PK2 was obtained by adding the mutated sequences α_R2S_; α_A9D_; α_A20T_; α_T24S_; α_R2S_,_A9D_; α_A20T_,_T24S_; α_R2S_,_A9D_,_A20T_,_T24S_; and α_E86G,A87T_ in equimolar concentration and in a 2:1 rate respect to *Bam*HI/ *Not*I linearized pJRoC30 plasmid and transformed *S. cerevisiae* cells using IVOE. Laccase activities from 1600 clones library were analysed by high-throughput screening with 3 mM ABTS and 50 mM Citrate Phosphate pH 3.0, and double checked by a first rescreening and second rescreening as it is described in Camarero and Co-workers [32].
V. CSM *N*-Gly58/59 and *N*-Gly68/69 libraries were obtained by combinatorial saturation mutagenesis over the second and third positions of 57 and 67 *N*-glycosylation sites of the α-factor preproleader (specifically of the optimised α_A9D,A20T_ leader). Degenerated sense and anti-sense primers (Arg-X-Ser/Thr) were used to replace the amino acid of the second position by all possible amino acid residues (except for Pro) while maintaining the *N*-glycosylation consensus sequence. CSM 86/87 library was obtained by combinatorial saturation mutagenesis at 86 and 87 positions of the optimised α_A9D,A20T_ leader, using codon degenerated sense and anti-sense oligos (Table. S2) to cover all possible 20 standard amino acid substitutions. The mutated α-factor leaders attached to ApL and PK2 laccases cloned in pJRoC30 were used to transformed *S. cerevisiae* cells. Up to 160 clones of each CSM *N*-Gly library (coverage at 95% of confidence based on GLUE-IT programme [76]) and 1,600 clones of each CSM 86/87 library (coverage at 90% of confidence) were analysed by high-throughput screening with 3 mM ABTS and 50 mM CP pH 3.0 and landscapes for the activities of the different clones were obtain for each library respecting the parental activity [32].

### Top-down, bottom-up and expression assays of other enzymes

Assays of expression were analysed in 96-well plates. Ten single colonies of every variant were selected and incubated in 50 μl of MM at 28 °C and 80% humidity to prevent evaporation in a humidity shaker (Minitron-INFORS). After 24 h 160 μl of SEM expression medium were added and incubated for 48 h at same conditions. Plates were centrifuged, 10 min, at 1000 g, 4 °C, and 20 μl of supernatant were transferred to a new plate. The replica plate was filled according to the enzyme as follow; laccases with 3 mM ABTS, 50 mM CP pH3; AAO with 2 mM *p*-methoxybenzyl alcohol, 100 mM phosphate buffer pH 6, Horseradish peroxidase (HRP) and 3 mM ABTS; VP with 3 mM ABTS, 100 mM tartrate buffer pH 3, 8 mM H_2_O_2_. After stirring plates were measured in kinetic mode at 418 nm for ABTS (ε418 = 36,000 M^−1^ cm^−1^), in SpectraMax M2 plate reader (Molecular Devices) and were normalized against the parental. OPE activity was measured as previously described [77]. BGL activity was determined with 5 mM *p*-nitrophenyl-β-d-glucopyranoside (pNPG) in 50 mM acetate buffer pH 4. The reaction was stopped after 10 min with sodium carbonate (2% w/v at the well) and measured at 410 nm.

### Enzyme production in flask

Three single colonies from parental and variants α-factor leaders were inoculated in 3 ml MM at 28 °C and 200 rpm. After 48 h cultures were diluted to OD600 = 0.3 and incubated until a final OD600 = 1. Thereafter, 27 ml EB medium was inoculated with 3 ml of preculture in 250 ml flasks and incubated for 96 h at 28 °C and 200 rpm. Every 24 h a 1 ml aliquot was extracted from the cultures to measure their growth (OD600) and laccase activity using 3 mM ABTS, 50 mM CP pH 3 in kinetic mode at 418 nm in SpectraMax M2 plate reader (Molecular Devices).

### Enzyme purification and specific activity

PK2 laccase with α_OPT_ or α_OPT Eα86T/Aα87N_ were grown in 1.2 l EB medium using 1 l flasks as described above. After 4 days of incubation, liquid extracts were filtrated (through 0.22 μm cut off membrane) and concentrated and ultra-diafiltrated using Pellicon tangential filtration membranes (Merck Millipore, Germany) and Amicon stirred cells (Merck Millipore, Germany), both with a 10 kDa cut off. Laccases were purified by FPLC in three anion exchange and an exclusion size chromatography steps: (i) HiPrep QFF 16/10 column in a 100 ml gradient of 0–40% elution buffer, (ii) HiTrap QFF 5 ml in a 100 ml gradient of 0–40% elution buffer, (iii) Mono Q HR 5/5 column in a 30 ml gradient of 0–25% elution buffer, and (iv) Superdex 75. All columns were purchased from GE Healthcare. Enzyme purification was confirmed by SDS-PAGE (12% acrylamide) stained. For specific activity the final protein concentration was calculated by nanodrop (A280 nm) and laccase activity using 3 mM ABTS, 50 mM CP pH 3 in kinetic mode at 418 nm in SpectraMax M2 plate reader (Molecular Devices). Deglycosylation by Endo H (Merck) was performed following the seller´s recommendations.

### DNA sequencing

The pJRoC30 plasmid containing enzymes were sequenced by MACROGEN, using the ExtFw sense and ExtRv antisense.

### Statistical analysis

R was use for the statistical comparison among means of ten replicates of every variant. After an Analysis of Variance, the Tukey’s range test was used to determine significant differences from a set of means. Tukey’s range test is a multiple comparison test and is applicable when there are more than two means being compared.

## Supplementary Information

Below is the link to the electronic supplementary material.

Supplementary file1 (PDF 1311 KB)

## Data Availability

The data generated or analysed during this study are included in this published article and its supplementary information files.
