# Supplementary material for: Design of an improved universal signal peptide based on the α-factor mating secretion signal for enzyme production in yeast
Source: Cell Mol Life Sci. 2021 Mar 9;78(7):3691–707. doi: 10.1007/s00018-021-03793-y (PMC8038962; doi:10.1007/s00018-021-03793-y)
Supplement: Supplementary file 1 — Supplementary file1 (PDF 1311 KB) [file 18_2021_3793_MOESM1_ESM.pdf]

SUPPLEMENTARY MATERIAL

Table S1. Sequences of the primers used for individual mutation of α-factor preproleader

ExtFw	5´CTGGGGTAATTAATCAGCGAAGCGATG3´
ExtRv	5´GAGCGTCCCAAAACCTTCTCAAGCAAG3´
R2S-Fw	5´ATGAGCTTTCCTTCAATTTTTACTGCTG3´
R2S-Rv	5´CAGCAGTAAAAATTGAAGGAAAGCTCAT3´
A9D-Fw	5´GGATCATAGGATCCATGAGATTTCCTTCAATTTTTACTGATGTT3´
A9D-Rv	5´AACATCAGTAAAAATTGAAGGAAATCTCATGGATCCTATGATCC3´
A20T-Fw	5´CTCCGCATTAGCTACTCCAGTCAACAC3´
A20T-Rv	5´GTGTTGACTGGAGTAGCTAATGCGGAG3´
T24S-Fw	5´GTCAACAGTACAACAGAAGATGAAACGG3´
T24S-Rv	5´CCGTTTCATCTTCTGTTGTACTGTTGAC3´
Q32H-Fw	5´GATGAAACGGCACATATTCCGGCTGAAGCT3´
Q32H-Rv	5´AGCTTCAGCCGGAATATGTGCCGTTTCATC3´
S42L-Fw	5´CGGTTACTTAGATTTAGAAGGGGATTTCG3´
S42L-Rv	5´CGAAATCCCCTTCTAAATCTAAGTAACCG3´
L44S-Fw	5´CGGTTACTCAGATTCAGAAGGGGATTTCGA3´
L44S-Rv	5´TCGAAATCCCCTTCTGAATCTGAGTAACCG3´
F48S-Fw	5´TAGAAGGGGATTCCGATGTTGCTG3´
F48S-Rv	5´CAGCAACATCGGAATCCCCTTCTA3´
S58G-Fw	5´GCTGTTTTGCCATTTTCCAACGGCACA3´
S58G-Rv	5´TGTGCCGTTGGAAAATGGCAAAACAGC3´
G62R-Fw	5´AACAGATTATTGTTTATAAATACTAC3´
G62R-Rv	5´GTAGTATTTATAAACAATAATCTGTT3´
E83D-Fw	5´GGGGTATCTCTCGATAAAAGAGAGG3´
E83D-Rv	5´CCTCTCTTTTATCGAGAGATACCCC3´
E86G-Fw	5´GAAGAAGGGGTATCTCTCGAGAAAAGAGGG3´
E86G-Rv	5´CCCTCTTTTCTCGAGAGATACCCCTTCTTC3´
A87T-Fw	5´CTCTCGAGAAAAGAGAGACTGAAGCT3´
A87T-Rv	5´AGCTTCAGTCTCTCTTTTCTCGAGAG3´
Revert H32Q-Fw	5´GATGAAACGGCACAAATTCCGGCTGAA3´
Revert H32Q-Rv	5´TTCAGCCGGAATTTGTGCCGTTTCATC3´
Revert S48F-Fw	5´CAGATTTAGAAGGGGATTTTGATGTTGCTGTTTTGCC3´
Revert S48F-Rv	5´GGCAAAACAGCAACATCAAAATCCCCTTCTAAATCTG3´
Revert G58S-Fw	5´GCTGTTTTGCCATTTTCCAACAGCACA3´
Revert G58S-Rv	5´TGTGCTGTTGGAAAATGGCAAAACAGC3´
Revert R62G-Fw	5´AATAACGGTTTATTGTTTATAAATACTACTATTGCCAGCATTGCT3´
Revert R62G-Rv	5´AGCAATGCTGGCAATAGTAGTATTTATAAACAATAAACCGTTATT3´
Double R2S-A9D-Fw	5´ATGAGCTTTCCTTCAATTTTTACTGATG3´
Double R2S-A9D-Rv	5´CATCAGTAAAAATTGAAGGAAAGCTCAT3´
Double E86G- A87T-Fw	5´AAAAGAGGGACTGAAGCTGAATTC3´
Double E86G- A87T-Rv	5´ GAATTCAGCTTCAGTCCCTCTTTT3´

Table S2. Primer sequences for enzyme cloning in pJRoC30 plasmid

NAT-AAO-FW	5´GAAGCAGAATTCGCCGATTTTGACTAC3´
NAT-AAO-RV	5´GTAGTCAAAATCGGCGAATTCTGCTTC3´
NAT-BGL2-FW	5´GAAGCAGAATTCGCCACAGCTGCTTTGG3´
NAT-BGL2-RV	5´CCAAAGCAGCTGTGGCGAATTCTGCTTC3´
NAT-BGL3-FW	5´GAAGCAGAATTCTACTCTCCTCCAGCTT3´
NAT-BGL3-RV	5´AAGCTGGAGGAGAGTAGAATTCTGCTTC3´
NAT-OPE-FW	5´GAAGCAGAATTCACAACCGTGAATGTAAACTA3´
NAT-OPE-RV	5´TAGTTTACATTCACGGTTGTGAATTCTGCTTC3´
PJROC30-AAO-RV	5´ATGCTCGAGCGGCCGCCTACTGATCAGCCTT3´
PJROC30-BGL2-RV	5´ATGCTCGAGCGGCCGCTTACAGGCATTGAGAGTA3´
PJROC30-BGL3-RV	5´ATGCTCGAGCGGCCGCTTAATGCCCAATCTTCAA3´
PJROC30-OPE-RV	5´ATGCTCGAGCGGCCGCTTAGATGCGGAAGATGCC3´
NatFinal-Fw	5´AGGGAAGCCGAAGCAGAATTC3´
NatFinal-Rv	5´GAATTCTGCTTCGGCTTCCCT3´
87Final-Fw	5´CTCTCGAGAAAAGAGAGACTGAAGCTGAATTC3´
87Final-Rv	5´GAATTCAGCTTCAGTCTCTCTTTTCTCGAGAG3´

Table S3. Sequences of primers for CSM. Degenerated residues are underlined

N-GLY58/59-FW	5´CCATTTTCCAAC<u>NNKWCT</u>AATAACGGG3´
N-GLY58/59-RV	5´CCCGTTATT<u>AGWMNN</u>GTTGGAAAATGG3´
N-GLY68/69-FW	5´AAT<u>NNKWCT</u>ATTGCCAGCATTGCT3´
N-GLY68/69-RW	5´AGCAATGCTGGCAAT<u>AGWMNN</u>ATT3´
CSM 86/87-FW	5´CTCGAGAAAAGA<u>NNKNNK</u>GAAGCTGAATTC3´
CSM 86/87-RV	5´GAATTCAGCTTC<u>MNNMNN</u>TCTTTTCTCGAG3´

Fig S1. Amino acid and nucleotide sequences of the α-factor preproleader from Invitrogen (α_{nat} in this work). Positions 90th and 91st referred to the *Eco*RI restriction site that was introduced to facilitate genetic engineering.

Position	1	2	3	4	5	6	7	8	9	10	11	12	13	14	15	16	17	18	19	20
Amino acid	M	R	F	P	S	I	F	T	A	V	L	F	A	A	S	S	A	L	A	A
Codon	atg	aga	ttt	cct	tca	att	ttt	act	gct	gtt	tta	ttc	gca	gca	tcc	tcc	gca	tta	gct	gct

Position	21	22	23	24	25	26	27	28	29	30	31	32	33	34	35	36	37	38	39	40
Amino acid	P	V	N	T	T	T	E	D	E	T	A	Q	I	P	A	E	A	V	I	G
Codon	cca	gtc	aac	act	aca	aca	gaa	gat	gaa	acg	gca	caa	att	ccg	gct	gaa	gct	gtc	atc	ggt

Position	41	42	43	44	45	46	47	48	49	50	51	52	53	54	55	56	57	58	59	60
Amino acid	Y	S	D	L	E	G	D	F	D	V	A	V	L	P	F	S	N	S	T	N
Codon	tac	tca	gat	tta	gaa	ggg	gat	ttc	gat	gtt	gct	gtt	ttg	cca	ttt	tcc	aac	agc	aca	aat

Position	61	62	63	64	65	66	67	68	69	70	71	72	73	74	75	76	77	78	79	80
Amino acid	N	G	L	L	F	I	N	T	T	I	A	S	I	A	A	K	E	E	G	V
Codon	aac	ggg	tta	ttg	ttt	ata	aat	act	act	att	gcc	agc	att	gct	gct	aaa	gaa	gaa	ggg	gta

Position	81	82	83	84	85	86	87	88	89	90	91
Amino acid	S	L	E	K	R	E	A	E	A	E	F
Codon	tct	ctc	gag	aaa	aga	gag	gct	gaa	gct	gaa	ttc

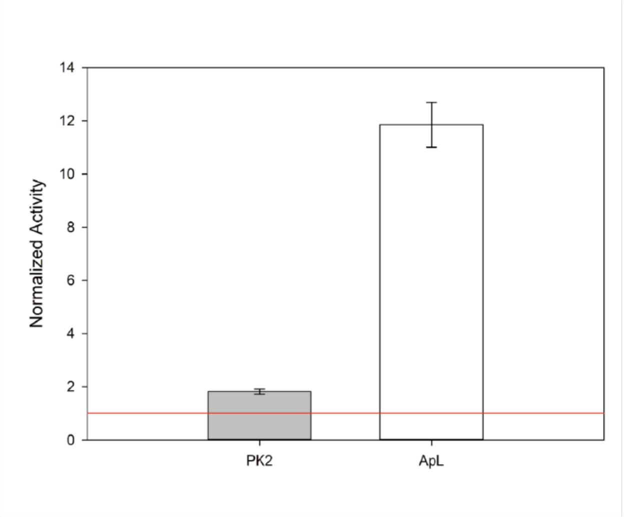

Fig S2. Laccase activities secreted by *S. cerevisiae* microcultures expressing PK2 (grey bars) or ApL (white bars) fused to α_{9H2} leader. Laccase activities were normalized to the activity of the corresponding parent type, α_{nat}-PK2 or α_{nat}-ApL (red line). Error bars correspond to the error propagation of ten replicates of each parent type or individual mutant

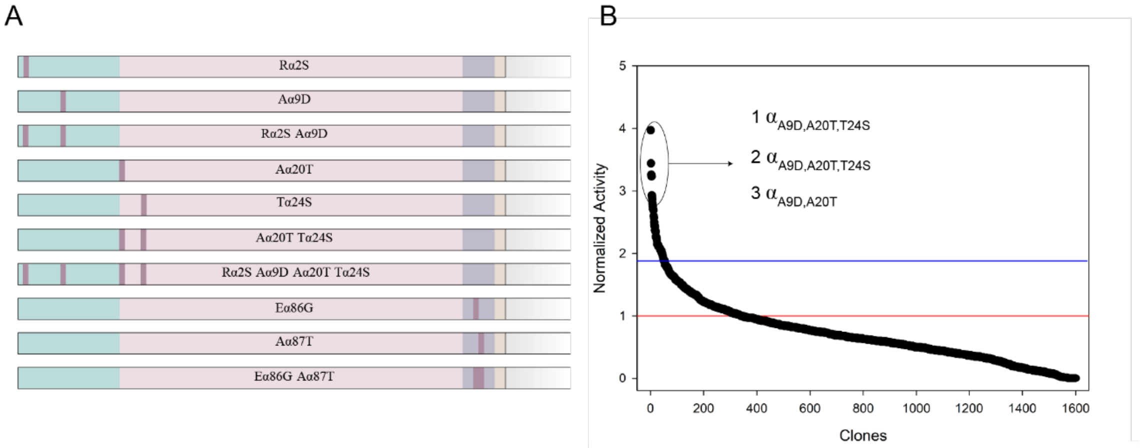

Fig S3. a Mutated α leaders (fused to PK2 laccase) selected for *in vivo* recombination in *S. cerevisiae*. **b** Activity landscape of the *in vivo* recombination library screened with ABTS pH 3; The activities of the different clones are normalised to the activity obtained with α_{nat} (red line). Laccase activity obtained with the construction $\alpha_{E86G,A87T}$-PK2 (blue line) was used as upper reference to select the fittest clones

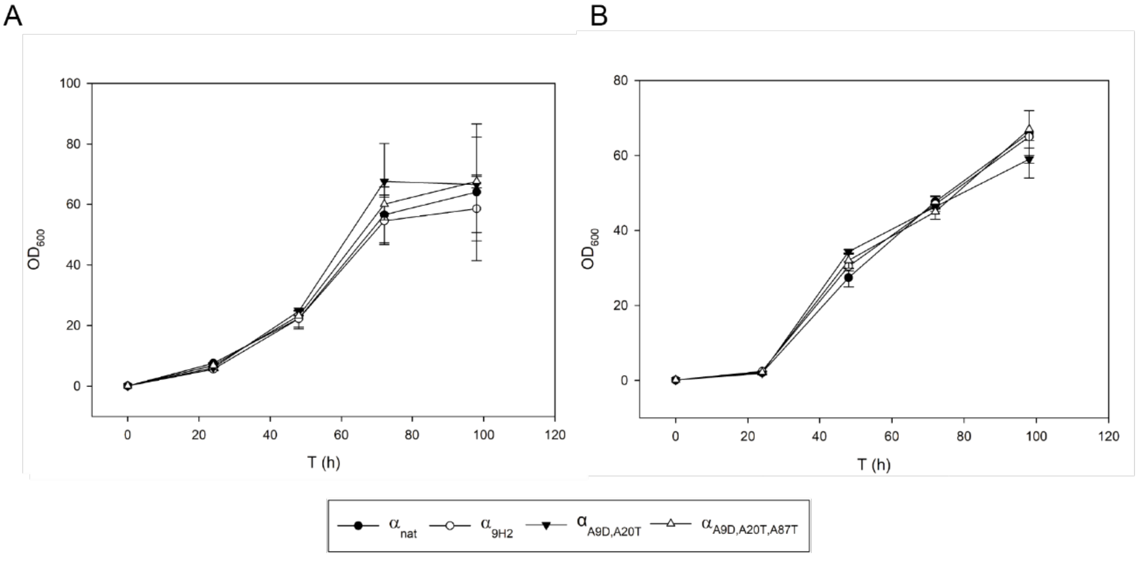

Fig S4. Optical densities (OD_{600}) of *S. cerevisiae* flask cultures expressing PK2 (**a**) or ApL (**b**) laccases with the best α-factor preproleaders obtained in the bottom-up ($\alpha_{A9D,A20T}$) and top-down ($\alpha_{A9D,A20T,A87T}$) strategies compared with α_{nat} and α_{9H2} leaders. Error bars indicate standard derivation of three flask replicates

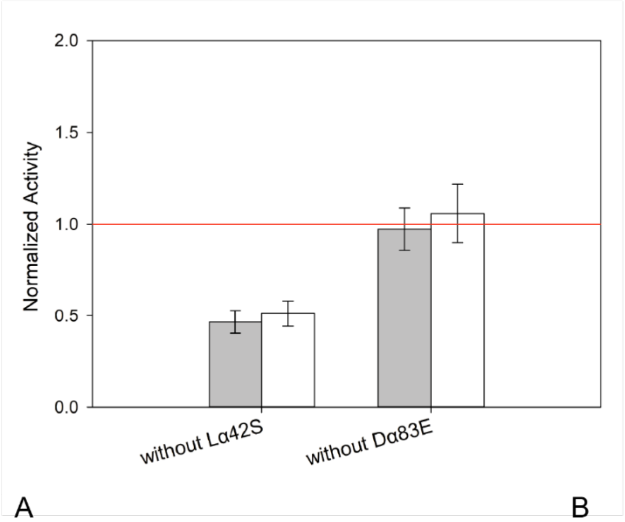

Fig S5. Laccase activities detected by *S. cerevisiae* microcultures expressing either PK2 (grey bars) or ApL (white bars) fused to $\alpha_{A9D,A20T}$ without Lα42S or Dα83E. Laccase activities were normalized to that of the corresponding parent type, $\alpha_{A9D,A20T}$-PK2 or $\alpha_{A9D,A20T}$-ApL (red line). Error bars correspond to the error propagation of ten replicates of each parent type or individual mutant

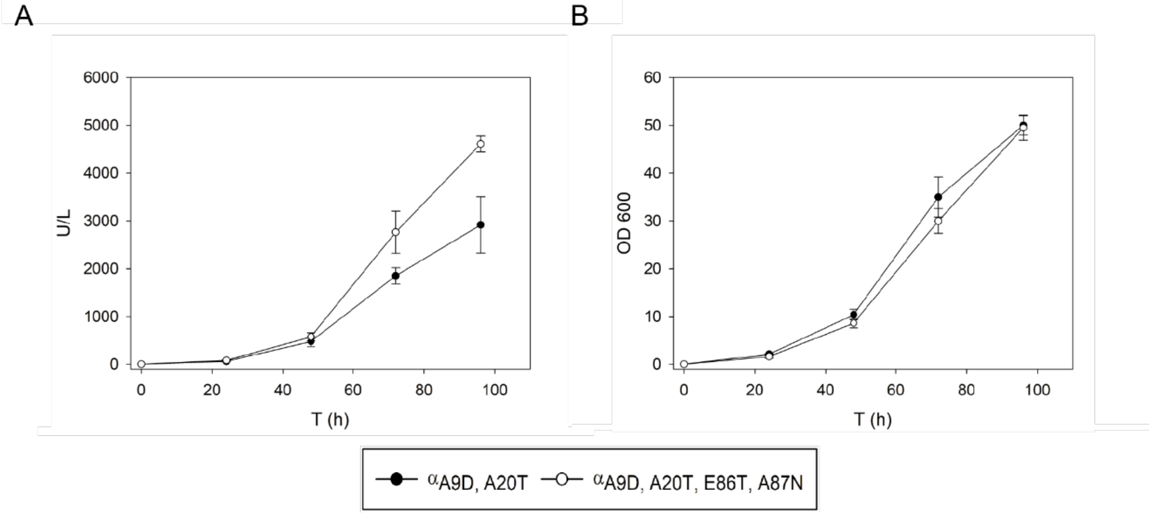

Fig S6. Flask production of PK2 laccase by *S. cerevisiae* using the mutated leaders α_{OPT} (black circle) or $\alpha_{OPT\ E\alpha86T/A\alpha87N}$ (white circle) as signal peptides. a Laccase activity (U/L) measured with ABTS pH 3. b Optical densities. Error bars indicate standard derivation of three flask replicates.

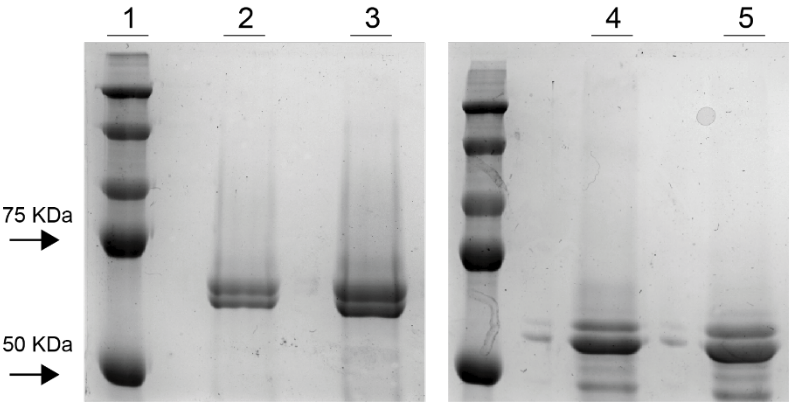

Fig S7. 10% SDS-PAGE of PK2 laccase produced by *S. cerevisiae* with α_{OPT} or $\alpha_{OPT\ E\alpha86T/A\alpha87N}$ as leaders, before (left) and after (right) deglycosylation with Endo H. Both gels were stained with Coomassie. The theorical molecular weight for PK2 is 53 KDa. Lanes: 1. Pre-stained protein Ladder (Biorad); 2. Purified PK2 laccase with α_{OPT} leader; 3. Purified PK2 laccase with $\alpha_{OPT\ E\alpha86T/A\alpha87N}$ leader 4. Purified PK2 laccase with α_{OPT} leader after Endo H treatment; 5. Purified PK2 laccase with $\alpha_{OPT\ E\alpha86T/A\alpha87N}$ leader after Endo H treatment.

	10 20 30 40 50 60 70 80
YPL187W S288C	MRFPSIFTAVLFAASSALAAPVNTTTEDETAQIPAEAVIGYLDLEGDFDVAVLPFSNSTNNGLLFINTTIASIAAKEEGVSLDKREAEA
YPL187W AWRI796	MRFPSIFTAVLFAASSALAAPVNTTTEDETAQIPAEAVIGYLDLEGDFDVAVLPFSNSTNNGLLFINTTIASIAAKEEGVSLDKREAEA
YPL187W BC187	MRFPSIFTAVLFAASSALAAPVNTTTEDETAQIPAEAVIGYLDLEGDFDVAVLPFSNSTNNGLLFINTTIASIAAKEEGVSLDKREAEA
YPL187W BY4741	MRFPSIFTAVLFAASSALAAPVNTTTEDETAQIPAEAVIGYLDLEGDFDVAVLPFSNSTNNGLLFINTTIASIAAKEEGVSLDKREAEA
YPL187W BY4742	MRFPSIFTAVLFAASSALAAPVNTTTEDETAQIPAEAVIGYLDLEGDFDVAVLPFSNSTNNGLLFINTTIASIAAKEEGVSLDKREAEA
YPL187W CBS7960	MRFPSIFTAVLFAASSALAAPVNTTTEDETAQIPAEAVIGYLDLEGDFDVAVLPFSNSTNNGLLFINTTIASIAAKEEGVSLDKREAEA
YPL187W CEN.PK	MRFPSIFTAVLFAASSALAAPVNTTTEDETAQIPAEAVIGYLDLEGDFDVAVLPFSNSTNNGLLFINTTIASIAAKEEGVSLDKREAEA
YPL187W CLIB215	MRFPSIFTAVLFAASSALAAPVNTTTEDETAQIPAEAVIGYLDLEGDFDVAVLPFSNSTNNGLLFINTTIASIAAKEEGVSLDKREAEA
YPL187W CLIB324	MRFPSIFTAVLFAASSALAAPVNTTTEDETAQIPAEAVIGYLDLEGDFDVAVLPFSNSTNNGLLFINTTIASIAAKEEGVSLDKREAEA
YPL187W D273-10B	MRFPSIFTAVLFAASSALAAPVNTTTEDETAQIPAEAVIGYLDLEGDFDVAVLPFSNSTNNGLLFINTTIASIAAKEEGVSLDKREAEA
YPL187W DBVPG6044	MRFPSIFTAVLFAASSALAAPVNTTTEDETAQIPAEAVIGYLDLEGDFDVAVLPFSNSTNNGLLFINTTIASIAAKEEGVSLDKREAEA
YPL187W EC1118	MRFPSIFTAVLFAASSALAAPVNTTTEDETAQIPAEAVIGYLDLEGDFDVAVLPFSNSTNNGLLFINTTIASIAAKEEGVSLDKREAEA
YPL187W FL100	MRFPSIFTAVLFAASSALAAPVNTTTEDETAQIPAEAVIGYLDLEGDFDVAVLPFSNSTNNGLLFINTTIASIAAKEEGVSLDKREAEA
YPL187W FY1679	MRFPSIFTAVLFAASSALAAPVNTTTEDETAQIPAEAVIGYLDLEGDFDVAVLPFSNSTNNGLLFINTTIASIAAKEEGVSLDKREAEA
YPL187W JK9-3d	MRFPSIFTAVLFAASSALAAPVNTTTEDETAQIPAEAVIGYLDLEGDFDVAVLPFSNSTNNGLLFINTTIASIAAKEEGVSLDKREAEA
YPL187W Kyokai7	MRFPSIFTAVLFAASSALAAPVNTTTEDETAQIPAEAVIGYLDLEGDFDVAVLPFSNSTNNGLLFINTTIASIAAKEEGVSLDKREAEA
YPL187W L1528	MRFPSIFTAVLFAASSALAAPVNTTTEDETAQIPAEAVIGYLDLEGDFDVAVLPFSNSTNNGLLFINTTIANIAAKEEGVSLDKREAEA
YPL187W LalvinQA23	MRFPSIFTAVLFAASSALAAPVNTTTEDETAQIPAEAVIGYLDLEGDFDVAVLPFSNSTNNGLLFINTTIASIAAKEEGVSLDKREAEA
YPL187W RM11-1a	MRFPSIFTAVLFAASSALAAPVNTTTEDETAQIPAEAVIGYLDLEGDFDVAVLPFSNSTNNGLLFINTTIASIAAKEEGVSLDKREAEA
YPL187W SEY6210	MRFPSIFTAVLFAASSALAAPVNTTTEDETAQIPAEAVIGYLDLEGDFDVAVLPFSNSTNNGLLFINTTIASIAAKEEGVSLDKREAEA
YPL187W SK1	MRFPSIFTAVLFAASSALAAPVNTTTEDETAQIPAEAVIGYLDLEGDFDVAVLPFSNSTNNGLLFINTTIASIAAKEEGVSLDKREAEA
YPL187W Sigma1278b	MRFPSIFTAVLFAASSALAAPVNTTTEDETAQIPAEAVIGYLDLEGDFDVAVLPFSNSTNNGLLFINTTIASIAAKEEGVSLDKREAEA
YPL187W UWOPS05 217 3	MRFPSIFTAVLFAASSALAAPVNTTTEDETAQIPAEAVIGYLDLEGDFDVAVLPFSNSTNNGLLFINTTIASIAAKEEGVSLDKREAEA
YPL187W VL3	MRFPSIFTAVLFAASSALAAPVNTTTEDETAQIPAEAVIGYXDLEGDFDVAVLPFSNSTNNGLLFINTTIAXIAAKEEGVSLDKREAEA
YPL187W W303	MRFPSIFTAVLFAASSALAAPVNTTTEDETAQIPAEAVIGYLDLEGDFDVAVLPFSNSTNNGLLFINTTIASIAAKEEGVSLDKREAEA
YPL187W X2180-1A	MRFPSIFTAVLFAASSALAAPVNTTTEDETAQIPAEAVIGYLDLEGDFDVAVLPFSNSTNNGLLFINTTIASIAAKEEGVSLDKREAEA
YPL187W Y55	MRFPSIFTAVLFAASSALAAPVNTTTEDETAQIPAEAVIGYLDLEGDFDVAVLPFSNSTNNGLLFINTTIASIAAKEEGVSLDKREAEA
YPL187W YJM339	MRFPSIFTTVLFAASSALAAPVNTTTEDETAQIPAEAVIGYLDLEGDFDVAVLPFSNSTNNGLLFINTTIASIAAKEEGVSLDKREAEA
YPL187W YJM789	MRFPSIFTAVLFAASSALAAPVNTTTEDETAQIPAEAVIGYLDLEGDFDVAVLPFSNSTNNGLLFINTTIASIAAKEEGVSLDKREAEA
YPL187W YPH499	MRFPSIFTAVLFAASSALAAPVNTTTEDETAQIPAEAVIGYLDLEGDFDVAVLPFSNSTNNGLLFINTTIASIAAKEEGVSLDKREAEA
YPL187W YPS128	MRFPSIFTAVLFAASSALAAPVNTTTEDETAQIPAEAVIGYLDLEGDFDVAVLPFSNSTNNGLLFINTTIASIAAKEEGVSLDKREAEA
YPL187W YPS163	MRFPSIFTAVLFAASSALAAPVNTTTEDETAQIPAEAVIGYLDLEGDFDVAVLPFSNSTNNGLLFINTTIASIAAKEEGVSLDKREAEA
YPL187W YS9	MRFPSIFTAVLFAASSALAAPVNTTTEDETAQIPAEAVIGYLDLEGDFDVAVLPFSNSTNNGLLFINTTIASIAAKEEGVSLDKREAEA

Fig S8. Alignment of α-factor preproleader from *S. cerevisiae* strains available in Saccharomyces Genome Database (SGD; www.yeastgenome.org).
